# Frequency Response of Pressure-Sensitive Paints under Low-Pressure Conditions

**DOI:** 10.3390/s21093187

**Published:** 2021-05-04

**Authors:** Miku Kasai, Daisuke Sasaki, Takayuki Nagata, Taku Nonomura, Keisuke Asai

**Affiliations:** Department of Aerospace Engineering, Graduate School of Engineering, Tohoku University, 6-6-01 Aramakiaza-Aoba, Aoba-ku, Sendai, Miyagi 980-8579, Japan; dainagon0830@gmail.com (D.S.); takayuki.nagata.d3@tohoku.ac.jp (T.N.); taku.nonomura.d5@tohoku.ac.jp (T.N.)

**Keywords:** pressure-sensitive paint, frequency response, low pressure

## Abstract

The characteristics of fast-response pressure-sensitive paints (PSPs) in low-pressure conditions were evaluated. Three representative porous binders were investigated: polymer-ceramic PSP (PC-PSP), anodized-aluminum PSP (AA-PSP), and thin-layer chromatography PSP (TLC-PSP). For each PSP, two types of luminophores, Pt(II) meso-tetra (pentafluorophenyl) porphine (PtTFPP) and tris(bathophenanthroline) ruthenium dichloride (Ru(dpp)${}_{3}$), were used as sensor molecules. Pressure sensitivities, temperature sensitivities, and photodegradation rates were measured and evaluated using a pressure chamber. The effect of ambient pressure on the frequency response was investigated using an acoustic resonance tube. The diffusivity coefficients of PSPs were estimated from the measured frequency response and luminescent lifetime, and the governing factor of the frequency response under low-pressure conditions was identified. The results of static calibration show that PC-PSP/PtTFPP, AA-PSP/Ru(dpp)${}_{3}$, and TLC-PSP/PtTFPP have high pressure sensitivities that exceed 4%/kPa under low-pressure conditions and that temperature sensitivity and photodegradation rates become lower as the ambient pressure decreases. Dynamic calibration results show that the dynamic characteristics of PSPs with PtTFPP are dependent on the ambient pressure, whereas those of PSPs with Ru(dpp)${}_{3}$ are not influenced by the ambient pressure. This observation indicates that the governing factor in the frequency response under low-pressure conditions is the lifetime for PC-PSP and TLC-PSP, whereas the governing factor for AA-PSP is diffusion.

## 1. Introduction

Flow-field measurement is important in the field of fluid dynamics to evaluate the performance of fluid machinery and clarify fluid dynamics phenomena. Among the physical quantity measurements, pressure measurements provide the distribution of aerodynamic loads required for fluid machinery design. Pressure measurements also provide information on important flow phenomena such as shock waves and flow separation. Pressure distribution is conventionally measured using pressure taps [1]. For the measurement of a detailed pressure distribution in a complex model, it is necessary to design a large number of pressure taps and a wind tunnel model that incorporates them. It is very difficult to measure multi-point pressure, especially with small or thin test models.

Pressure-sensitive paints (PSPs) are used as a tool to measure the pressure distribution on a model surface [2] in a wind tunnel experiment. A PSP is a pressure sensor that uses photochemical reactions and is composed of dye molecules and a binder that adsorbs the dye to a model surface. When a PSP is applied to an object to be measured and illuminated with light of an appropriate wavelength, the dye is excited and emits luminescence with an intensity that corresponds to the ambient oxygen concentration. It is possible to indirectly measure the ambient pressure by measuring the luminescence emission of a PSP with an optical sensor. Since each dye molecule coated on a model surface plays the role of a sensor, the use of a PSP is a wide-range, high-resolution, and nonintrusive pressure measurement technique compared to the conventional measurement methods where pressure taps are used.

PSPs have also been developed for pressure measurements in low-oxygen-concentration environments. Asai et al. [3] developed a PSP using poly[1-trimethylsilyl)-1-propyne] (poly(TMSP)) [4], which has the highest oxygen permeability of 7700 cm${}^{3}$(STP) cm/($10^{10}$ cm${}^{2}$ s cm Hg) for cryogenic and unsteady pressure measurements. The poly(TMSP)-based PSP was applied to a wing surface for low-density wind tunnel tests [5,6,7]. However, polymer-based PSPs do not have sufficient frequency responses to measure high-frequency pressure fluctuations.

In recent years, research on PSP for the measurement of unsteady pressures has progressed rapidly [8,9,10]. Fast-response PSPs have been applied to various atmospheric pressure environments. A thin-layer chromatography (TLC) plate [11], which is a porous material, was first applied as a binder to a PSP for the measurement of unsteady pressure fluctuations. The response time of the TLC-PSP developed by Baron et al. [11] was at least as fast as 25 µs. The TLC-PSP was applied to measure the pressure fluctuations of air-jet impingement and shock-propagation in a shock tube [12,13], and the pressure distribution of shock wave diffraction [14]. An anodized-aluminum PSP (AA-PSP) developed by Asai [15] that uses an oxide film formed by anodization on an aluminum surface as a binder is applicable to measurements of unsteady pressure distributions. Sakaue [16] developed an AA-PSP that can respond to pressure step changes in approximately 35 µs with a 90% rise time. Numata et al. [17] clarified that phosphoric acid has a larger pore size than that used for the conventional AA-PSP and improves the response time up to 0.35 µs. AA-PSPs were applied to wind tunnel tests to visualize unsteady pressure distributions [18,19,20]. Nagata et al. [21] investigated the pressure distribution of a circular cylinder using a low-density wind tunnel with the AA-PSP under low-pressure conditions.

AA-PSPs have restrictions such as the model materials and model shapes to which it can be applied; therefore, a fast-response and sprayable PSP was desired. Scroggin et al. [22] first developed a binder that consists of a polymer and solid particles. Gregory et al. [23] further developed the sprayable porous binder by improving the binder developed by Scroggin et al. [22]. Sugioka et al. [24,25] more recently applied a polymer-ceramic PSP (PC-PSP) with a low arithmetic surface roughness of 0.5 µm and a cut-off frequency of 3 kHz to transonic wind tunnel tests of a NASA Common Research Model and measured buffet phenomena on a wing surface. As a result, the footprint of an oscillating shock wave was clearly visualized and the dominant frequencies of the pressure fluctuation due to buffet phenomena were clarified. The response time of a PSP with mesoporous silica was developed by Peng et al. [26] to a step pressure input of 100 µs, and its photodegradation rate was 0.4%/min. Egami et al. [27,28] improved the response time of a sprayable PC-PSP using tris(bathophenanthroline) ruthenium dichloride (Ru(dpp)${}_{3}$) to less than 10 µs, which is comparable to that of AA-PSPs.

The application of PSPs to measurements of unsteady low-pressure conditions poses two major issues. One of these issues is pressure sensitivity. The ambient test pressure in a low-pressure environment is less than that of a typical wind tunnel test under atmospheric pressure. The pressure fluctuation due to a flow is so small that a PSP applied in such conditions is required to have high pressure sensitivity. However, the low pressure causes a decrease in the pressure sensitivity. Nagata et al. [29] introduced a localized Stern–Volmer coefficient, which was then utilized to evaluate the ambient pressure dependence of an intensity ratio of PSP emissions against the pressure change normalized with respect to the ambient pressure [30]. The localized Stern–Volmer coefficients of conventional polymer-based PSP, the PC-PSP using Pt(II) meso-tetra (pentafluorophenyl) porphine (PtTFPP), and of the AA-PSP decrease with the pressure. Therefore, it is difficult to measure the pressure distribution on a model surface under low-pressure conditions.

The second major issue is whether the response time is sufficiently fast to follow phenomena in time-resolved measurements under low-pressure conditions. There have been various studies on the response time of a PSP under atmospheric pressure. The gas diffusion phenomenon in the PSP binder is considered to be a rate-determining factor in the response time of a PSP. A model that expresses the gas diffusive process with a one-dimensional diffusion equation agrees with the experimental results [31]. On the other hand, when a binder with sufficiently high gas diffusivity such as an AA-PSP is used, the emission lifetime of the PSP dye may become non-negligible with respect to the time scale of gas diffusion in the PSP binder. Sugimoto et al. [32] clarified that the governing factor of frequency characteristics is the emission lifetime by determination of the transfer function for the PC-PSP. Similarly, the frequency characteristics of an AA-PSP using Ru(dpp)${}_{3}$ were suggested to depend on both the emission lifetime and diffusion of oxygen into the binder. Kameda et al. [33] formulated a time-response model of PSPs that incorporates the effects of the dye emission lifetime for minute-amplitude sinusoidal pressure fluctuations. Pandey and Gregory [34] introduced a two-layer responsive model that considers an attenuation of excitation light, a so-called hiding factor, in the binder (e.g., a PC-PSP), in addition to the emission lifetime of the PSP. Nonomura and Asai [35] derived an analytical formulation/approximation for the harmonic responses of two layers of oxygen diffusion and luminescence quenching with the effect of luminescence lifetime taken into account. The luminescent lifetime of a PSP is long under low-pressure conditions; therefore, the frequency response characteristics of the PSP may decrease if the emission lifetime is dominant in the frequency response characteristics of the PSP. However, it should be noted that all of these evaluations were conducted in an atmospheric pressure environment, and the influences of the ambient pressure on the response time of the PSPs have yet to been clarified. It is necessary to clarify the governing factor of the luminescence response of PSPs under low-pressure conditions for unsteady pressure measurements using a PSP in a low-pressure environment.

In the present study, the static and dynamic characteristics of various PSPs were investigated toward the development of a PSP for time-resolved measurements under a low-pressure environment. Furthermore, the governing factors of the frequency response under low-pressure conditions were considered with respect to the magnitude of the PSP lifetime and the diffusivity coefficients of the PSPs.

## 2. Experimental Setup and Analytic Method

### 2.1. Materials and Preparation

**Figure 1:**
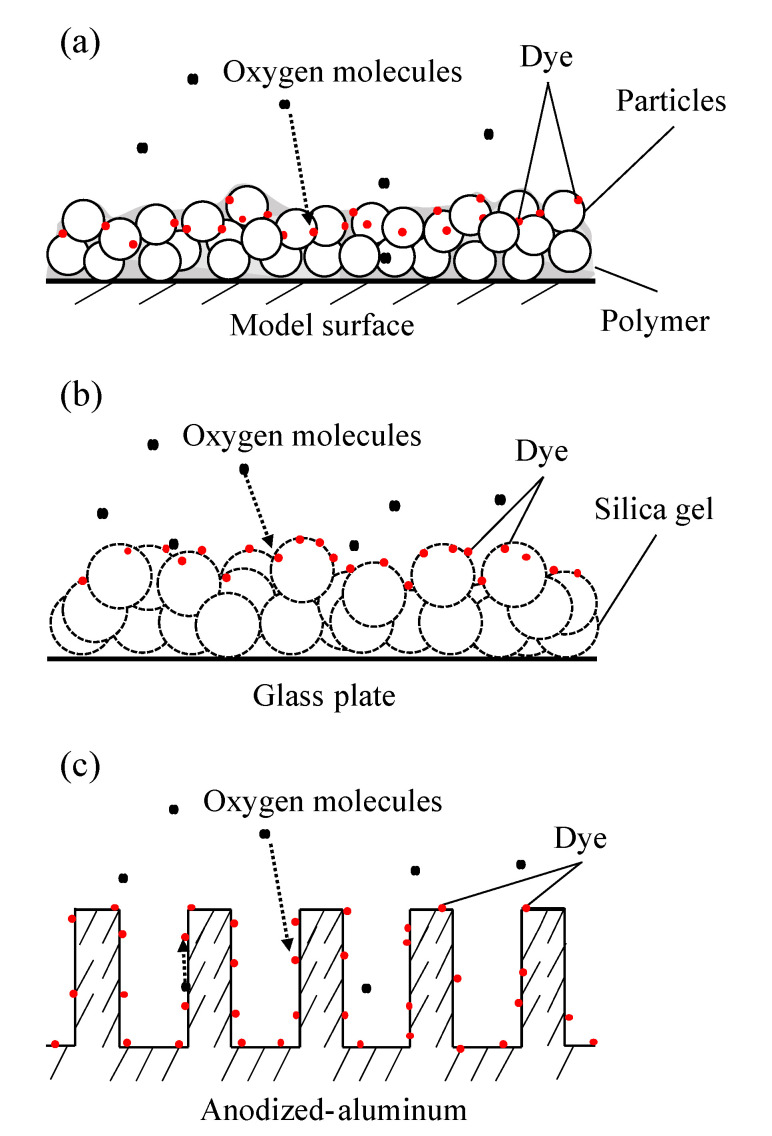
Schematic view of binder structure: (**a**) PC-PSP, (**b**) AA-PSP, and (**c**) TLC-PSP. (**a**,**c**) are taken from Sakaue et al. [36] and Sugioka et al. [37], respectively.

Figure 1 and Table 1 show three types of porous binders commonly used as fast-response PSPs that were evaluated: PC-PSP, AA-PSP, and TLC-PSP. PC-PSP is composed of particles with diameters of $10^{1}$–$10^{2}$ nm, and TLC-PSP is composed of silica gel with a particle diameter of $10^{4}$ nm with pore sizes of $10^{0}$ nm. AA-PSP has a porous structure with pores with size on the order of $10^{1}$–$10^{2}$ nm.

Table 2 shows the conditions under which samples were prepared. The dyes PtTFPP (PtT975, Frontier Scientific) and Ru(dpp)${}_{3}$ (GFS Chemicals) were applied to each of three porous binders.

#### 2.1.1. Polymer-Ceramic PSP

A sample coupon of the PC-PSP was prepared based on a method presented by Gregory et al. [38]. A dispersant (D-3005, Rohm and Haas) and titanium oxide (TiO${}_{2}$; TIO12PB, Kojundo Chemical Laboratory) with an average particle diameter of 500 nm were mixed in distilled water with a mixing ratio of dispersant:TiO${}_{2}$:distilled water weight ratio of 12 mg:1.72 g:1 g. Then, 10 mm diameter glass spheres were placed in the prepared ceramic slurry and mixed thoroughly with a ball mill. A polymer emulsion (Primal HA-8, Rohm and Haas) was added to the ceramic slurry at a weight fraction of 3–4% of the ceramic slurry. After further stirring of this polymer-ceramic binder, it was painted on the surface of a sample plate with a spray gun and dried for one day. The dye solvents were prepared by dissolving PtTFPP in toluene at the mixing ratio of PtTFPP:toluene = 4 mg:20 mL and Ru(dpp)${}_{3}$ dissolved in dichloromethane at a mixing ratio of Ru(dpp)${}_{3}$:dichloromethane = 14 mg:20 mL. These dye solvents were then sprayed over the precoated binder surface.

#### 2.1.2. Anodized-Aluminum PSP

Sample coupons of the AA-PSP were prepared according to the method proposed by Sakaue and Sullivan [39]. In the present study, anodizing oxidation was performed at a current density of 10.0 mA/cm${}^{2}$ while the temperature of the dilute sulfuric acid solution was maintained at 283 K by stirring. This method resulted in pore diameters for the AA-PSP on the order of 10${}^{1}$ to 10${}^{2}$ nm [39]. The adsorption behavior of AA-PSPs varies with the dye. Therefore, the adsorption method was modified for each dye as described below. In the case of PtTFPP, 8 mg of PtTFPP was mixed with 20 mL of hexane and a dye solvent was formed. The temperature of the dye solvent was set to 313 K and the anodized-aluminum coupon was immersed in the dye solvent for 1 min. Sample coupons were then dried thoroughly using a desiccator. A dye solution for Ru(dpp)${}_{3}$ was prepared by mixing 14 mg of Ru(dpp)${}_{3}$ with 20 mL of dichloromethane. The anodized-aluminum coupons were immersed in the dye solvent for approximately 10 s. Excess dye adhering to the surface was removed by dipping into dichloromethane. Finally, the coupons were again dried thoroughly using a desiccator.

#### 2.1.3. Thin-Layer Chromatography PSP

In the present study, commercially available TLC plates (Merck, TLC Silica gel 60) were used as the binder for the TLC-PSP [13]. The pore diameter of this silica gel is 60 Å, and the particle diameter is approximately 10 µm. The dye was adsorbed on the TLC binder after spraying with a spray gun. The dye solvents were prepared by dissolving PtTFPP in toluene at a ratio of PtTFPP:toluene = 4 mg:20 mL and Ru(dpp)${}_{3}$ dissolved in dichloromethane at the same mixing ratio as the PC-PSP at Ru(dpp)${}_{3}$:dichloromethane = 14 mg:20 mL.

### 2.2. Static Calibration

**Figure 2:**
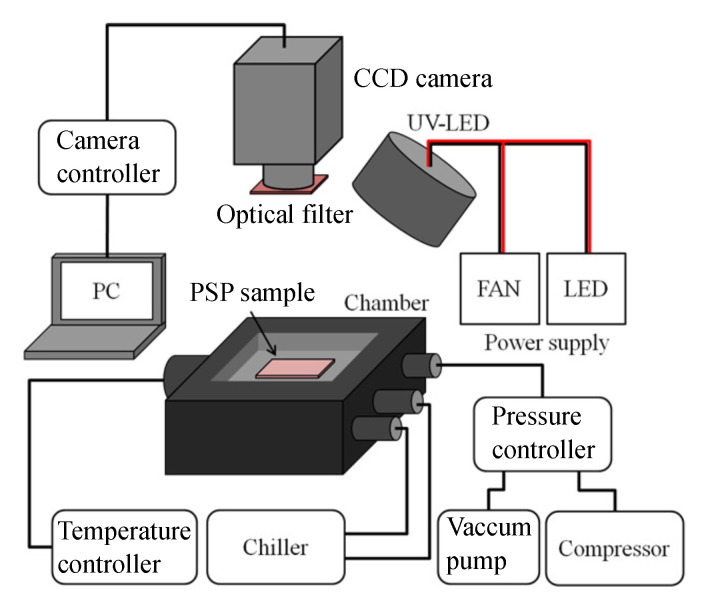
Schematic illustration of the experimental setup for static calibration.

The static characteristics of PSPs were obtained using a calibration system, as schematically shown in Figure 2. The sample coupons were placed in a calibration chamber where the pressure and temperature could be controlled. An ultraviolet (UV) LED (LEDH576-395, Hamamatsu Photonics) with a central wavelength of 395 nm was used as the excitation light source. The distance between the sample and the LED was set to approximately 300 mm, and the power output of the LED excitation source was 10 µW to 125 µW depending on the quantum yield and pressure. The luminescence intensity of the sample coupon was detected by a 16-bit CCD camera (ORCA II-BT 1024, Hamamatsu Photonics). A 50 mm focal length lens (Nikkor 50 mm f1.2, Nikon) was attached to a camera with a 560 nm long-pass filter (O-56, Hoya Candeo Optronics). Analysis was performed on an image of each sample in the range of 40×40 pixels, and the standard deviation was used as the error bar of each graph.

The pressure inside the chamber *P* and the temperature of the sample coupon *T* were varied in the range of 1–100 kPa and 278–303 K, respectively. The luminescence intensity *I* under each condition was normalized with respect to the intensity under two reference conditions of $P_{\mathrm{ref}}=10$ and 100 kPa at $T_{\mathrm{ref}}=293$ K. The intensity ratios were fitted to Equation (1) given by the Stern–Volmer relationship and Equation (2).

(1) $$\frac{I_{\mathrm{ref}}}{I}=A_{1}\frac{P}{P_{\mathrm{ref}}}+A_{2}.$$

(2) $$\frac{I}{I_{\mathrm{ref}}}=B_{1}T^{2}+B_{2}T+B_{3}.$$

The gradient of Equation (1) and the absolute value of the gradient of Equation (2) at each reference condition were determined as the pressure sensitivity $S_{P}$ and the temperature sensitivity $S_{T}$:

(3) $$S_{P}=\frac{\partial \left(I_{\mathrm{ref}}/I\right)}{\partial \left(P/P_{\mathrm{ref}}\right)}÷P_{\mathrm{ref}}\times 100=\frac{100A_{1}}{P_{\mathrm{ref}}},$$

(4) $$S_{T}=\left|\frac{\partial \left(I/I_{\mathrm{ref}}\right)}{\partial T}\right|\times 100=\left|2B_{1}T+B_{2}\right|\times 100.$$

The localized Stern–Volmer coefficient introduced by Nagata et al. [29] as a new evaluation index was calculated. The localized Stern–Volmer coefficient is the pressure evolution of the normalized sensitivity, which is the local gradient of the Stern–Volmer curve:

(5) $$B_{\mathrm{local}}\equiv \frac{\partial \{I\left(P_{\mathrm{ref}}\right)/I\left(P\right)\}}{\partial (P/P_{\mathrm{ref}})}.$$

The photodegradation rates of the PSPs were evaluated. A PSP sample was continuously irradiated with the excitation light for 30 min under constant conditions of P=1, 10, and 100 kPa at T=293 K. The distance between the sample and the LED was set to approximately 300 mm, and the output power of the LED excitation source was set to approximately 40 µW under all sample and pressure conditions in the photodegradation rates measurement. The photodegradation rate $I_{d}$ is defined as the rate of decrease in the normalized intensity over 30 min as expressed in Equation (6).

(6) $$I_{d}=-\left(1-\frac{I_{t=30\;\min}}{I_{t=0\;\min}}\right)\frac{1}{30}\times 100\;\left[\%/\min\right],$$

where $I_{t=0\;\min}$ and $I_{t=30\;\min}$ are the luminescence intensities at 0 and 30 min, respectively.

### 2.3. Dynamic Calibration

**Figure 3:**
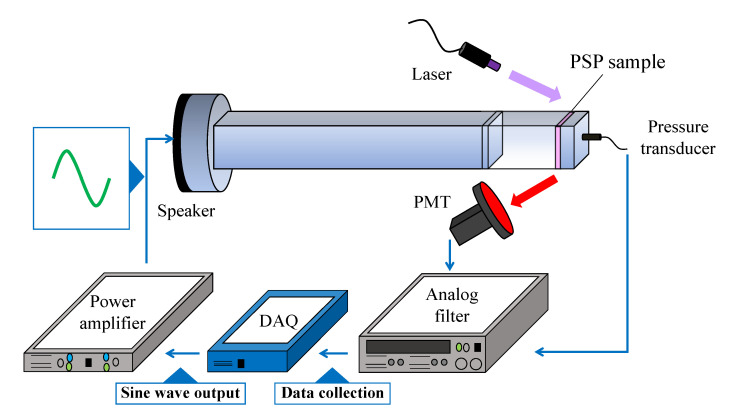
Schematic illustration of a resonance tube.

The dynamic characteristics of PSPs were investigated using a frequency response test with an acoustic resonance tube as shown in Figure 3 [32]. This acoustic resonance tube can generate sinusoidal pressure fluctuations on the order of kilopascals in the frequency range of 0.15–10 kHz. The end of the acoustic resonance tube was capped by a PSP sample plate and a cap with a pressure transducer in its center. Two types of pressure transducers were applied, depending on the pressure in the resonance tube: a CCQ-093-5A (Kulite) in the range of 5–10 kPa and an XCL-152-5SG (Kulite) in the range of 20–100 kPa. A temperature-measuring resistor (R060-39, Chino Corporation) and a Peltier device (FPH1-12706AC, Fujita Corporation) were installed on the back of the sample plate, and the temperature of the sample plate could be controlled by a Peltier controller (TD-1000A, Cell System Corporation).

The PSP sample coupon was excited with a UV laser (RV-1000TH, Ricoh, Yokohama, Japan), the wavelength of which was 400 nm. The distance between the sample and the laser was set to approximately 400 mm, and the power output of the laser was changed from 90 µW to 20 mW depending on the quantum yield and pressure. The emission from the sample was measured using a photomultiplier tube (PMT; H5784-02, Hamamatsu Photonics). A 560 nm long-pass filter (O-56, Hoya Candeo Optronics) was placed in front of the PMT. The pressure was measured at the same time as the PSP measurement with the pressure transducer installed in the center of the PSP sample. The PMT signal and the pressure transducer signal were acquired simultaneously with a data acquisition (DAQ) device (USB-6251, National Instruments). The output part has a speaker. One of the speakers is a low-frequency speaker (TU-750, TOA Corporation) and the other is a high-frequency speaker (RX22, Peavey). Low-frequency loudspeakers were used at 0.15–3 kHz (low-frequency loudspeaker: 0.15, 0.27, 0.39, 0.512, 0.65, 0.942, 1.216, 1.5, 1.928, 2.334, and 2.77 kHz.) On the other hand, the range of measurements with a high-frequency loudspeaker was set to 0.5–10 kHz (high-frequency loudspeaker: 0.586, 0.908, 1.184, 1.482, 1.934, 2.378, 2.784, 3.216, 3.814, 4.428, 5.02, 6.08, 6.95, 8.466, and 9.748 kHz.) The number of input cycles from the power amplifier (CP600, Classic Pro) to the speaker was $4\times 2^{10}$ cycles. The amplitude of the output voltage from the power amplifier to the speaker was approximately 0.125 to 0.5 W depending on the frequency. The pressure in the acoustic resonance tube, which is the output section of the speaker, was controlled by a pressure controller (DPI 515 Digital Pressure Controller, Druck).

The recorded signals of the PMT were then converted to pressure using an in-situ calibration result of the PSP at the lowest frequency (0.15 kHz). The recorded signals of the pressure transducer were converted into pressure data using an a-priori calibration result. The gain and phase delay of the PSP signal were calculated by comparing the amplitude and phase of the signals obtained by the pressure transducer and PSPs. The data analysis methods have been previously detailed [32]. The cut-off frequency, which is the frequency at which the gain attenuation is −3 dB, was used as an index of the frequency response of the PSPs.

**Figure 4:**
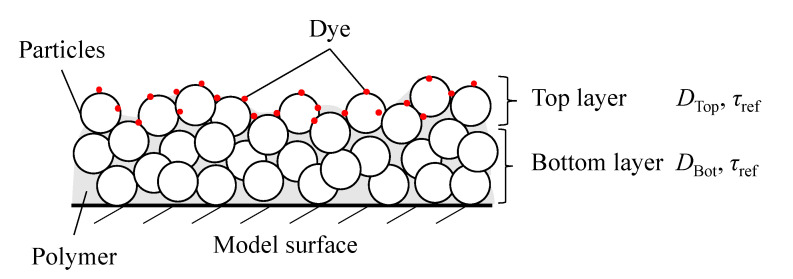
Schematic illustration of the two-layer model.

The diffusivity coefficient *D* and the hiding factor *c* of a PSP were estimated from the obtained gain and phase delays by fitting the frequency response of the two-layer PSP model proposed by Nonomura and Asai [35]. First, the hiding factor was estimated using the frequency response of all pressure cases, assuming a common diffusivity coefficient. The hiding factor of the PC-PSP was assumed to be constant in all pressure cases. Second, the diffusivity coefficient was estimated using the obtained hiding factor. Constraints in parameter estimation were different only for the PC-PSP from the other paints. The hiding factor was considered only for the PC-PSP. In the case of a two-layer structure in which a dye solvent is painted on the PC binder layer in the PC-PSP, the dye solvent merges into the PC binder layer, and the dye enters between the ceramic particles. In addition, two diffusivity coefficients, $D_{\mathrm{Top}}$ and $D_{\mathrm{Bot}}$, were approximated for top and bottom layers, respectively, because the PC-PSP used in the present study has a two-layer structure as shown in Figure 4. The thickness of the top layer is defined as the standard deviation (STD) of the paint thickness as defined by Pandey and Gregory [34]. Diffusivity coefficients were estimated under the constraint that the diffusivity coefficient of the top layer is larger than that of the bottom layer. When the dye was adsorbed on binders such as AA-PSP and TLC-PSP, the hiding factor was approximated as zero. The diffusivity coefficients of the TLC-PSP and AA-PSP were evaluated with the assumption that the thickness of the second layer was zero.

The harmonic pressure response measured for $P_{\omega }$ compared with a low-frequency approximation $P_{\omega ,\mathrm{ideal}}$, $P_{\omega }/P_{\omega ,\mathrm{ideal}}$, was firstly calculated from the obtained gain and phase delay of the PSP signal, and the frequency response data were approximated to the response model by changing the diffusivity and the hiding factor in the model. The STD of $P_{\omega }/P_{\omega ,\mathrm{ideal}}$ was calculated from the STD for the gain and phase delay by the same measurement. The approximation was performed with the gradient descent: each parameter was optimized to minimize the squared Frobenius norm of the difference between the experimental value and the model value of $P_{\omega }/P_{\omega ,\mathrm{ideal}}$. It should be noted that a reasonable optimization was realized with the small computational cost of the simple calculation of the model [35]. Here, the squared Frobenius norm was calculated after multiplying the difference between the model and the experimental value $P_{\omega }/P_{\omega ,\mathrm{ideal}}$ by the reciprocal of STD, which corresponds to the reliability of the data, as a weighting function. The values shown in Table 3 are given as the initial values of the optimization using the gradient descent for each binder. The diffusivity coefficient and hiding factor obtained at 100 kPa for each binder were given as initial values under the lower pressure conditions. The iterative calculation was stopped when a residual, which is the difference between the values of an objective function at a previous and a current step, was smaller than $10^{-10}$. In this study, the cut-off frequency was calculated as that at which the gain of the model approximated from the frequency response would be −3 dB.

**Figure 5:**
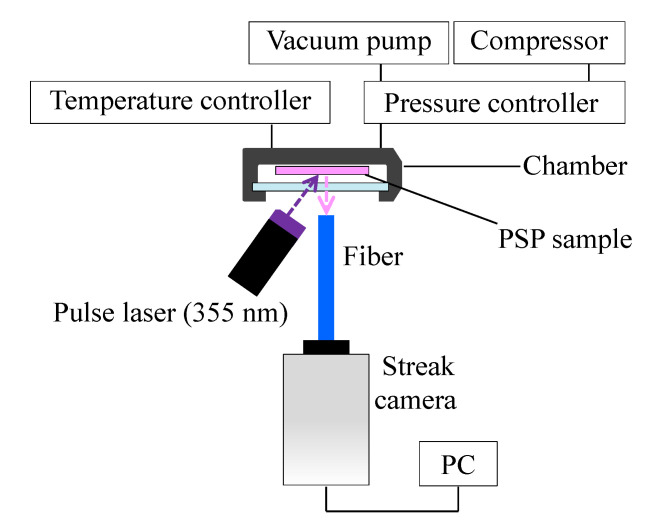
Schematic illustration of the experimental setup for lifetime measurements.

In the lifetime measurement, a PSP was excited using an ultraviolet laser with a single wavelength of 355 nm using the lifetime measurement system shown in Figure 5. The fall time of the laser pulse was 5 ns. The power output of excitation was 30 W. The lifetime measurement result is affected by the fall time of the excitation light source; therefore, a pulse laser with a short fall time was used. The light emission of the PSP was captured by a streak camera with an optical fiber installed in front of the sample coupon. A 370 nm long-pass filter was installed between the streak camera and the fiber. The temperature of the PSP sample coupon was controlled to 293 K with a Peltier module and the pressure was controlled to 1, 5, 10, 20, 50, and 100 kPa.

The obtained emission response curve was approximated by an exponential function (N=1–3) shown in Equation (7):

(7) $$I=\sum_{a}^{N}A_{n}\exp\left(-\frac{t}{\tau_{n}}\right),$$

where the minimum *N* with a coefficient of determination of 0.99 or more was adopted. The weighted mean lifetime was calculated from the obtained time constants [40] using Equation (8):

(8) $$\tau_{L}=\frac{\sum_{n=1}^{N}A_{n}\tau_{n}}{\sum_{n=1}^{N}A_{n}}.$$

## 3. Results

### 3.1. Static Characteristics

**Figure 6:**
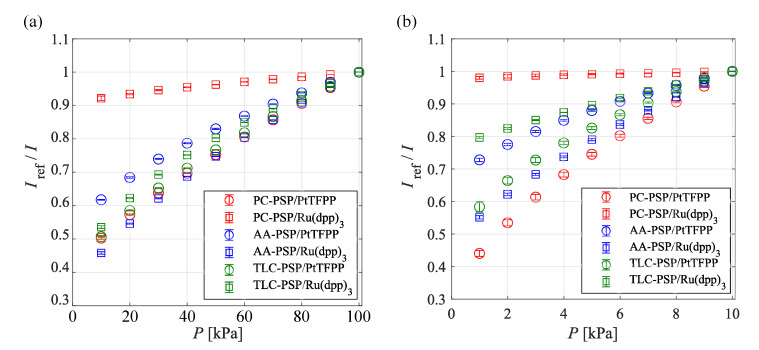
Pressure characteristics of PSPs in the range of (**a**) 10–100 kPa and (**b**) 1–10 kPa at 293 K.

Pressure calibration curves for each PSP in different pressure ranges are shown in Figure 6. The temperature of the sample coupon was fixed at 293 K. Figure 6a shows that the slope of the Stern–Volmer plot differs according to the binder and dye in the range of 10–100 kPa. The gradient of the Stern–Volmer curve for the PC-PSP is significantly influenced by the dye. The pressure sensitivity of PC-PSP/Ru(dpp)${}_{3}$ was the lowest, and that of PC-PSP/PtTFPP was the highest of all the PSPs investigated in this study. The influence of the dye on the pressure sensitivity is relatively small in the case of the AA-PSP and the TLC-PSP. In the range of 10–100 kPa, AA-PSP/Ru(dpp)${}_{3}$ had the highest pressure sensitivity of all the PSPs investigated in this study. Figure 6b shows the Stern–Volmer curve in the range of 1–10 kPa. The gradient of the Stern–Volmer curve was reduced and the difference in the dye had a larger effect on the pressure sensitivity in the range of 1–10 kPa. The pressure sensitivity of PC-PSP/Ru(dpp)${}_{3}$ was the lowest in the range of 1–10 kPa, similar to the pressure sensitivity in the range of 10–100 kPa, whereas PC-PSP/PtTFPP had the highest pressure sensitivity of all the PSPs investigated. The dye with the higher pressure sensitivity for each binder was the same as that in all the pressure ranges and PSPs that were investigated in the present study. Except for the AA-PSP, the PSPs with PtTFPP had higher pressure sensitivity than the PSPs using Ru(dpp)${}_{3}$.

**Figure 7:**
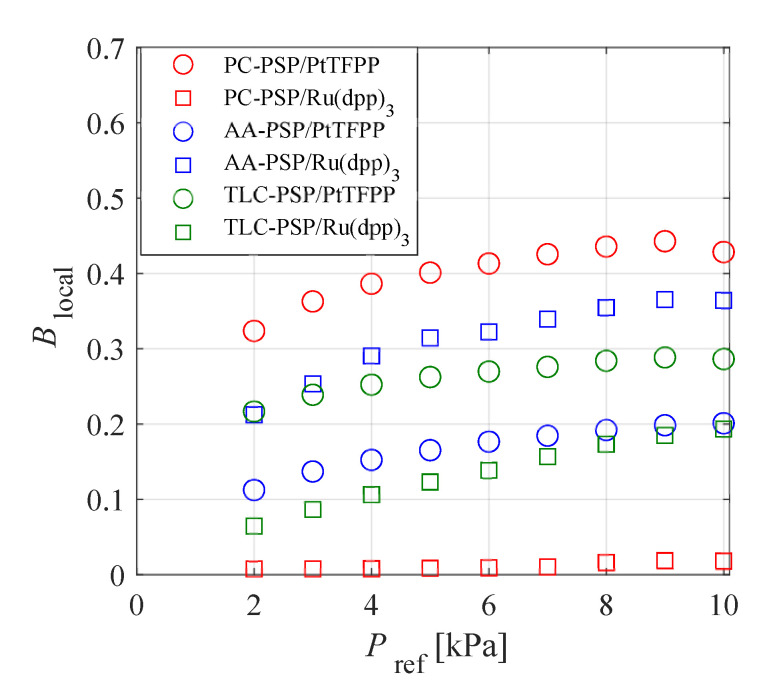
Localized pressure sensitivity as a function of the ambient pressure in the range of 1–10 kPa at 293 K.

Figure 7 shows the localized Stern–Volmer coefficient $B_{\mathrm{local}}$ calculated from Equation (5) plotted against $P_{\mathrm{ref}}$ in the range of $P_{\mathrm{ref}}\leq 10$ kPa. This figure indicates that the pressure sensitivity of each PSP in the low-pressure range was different. The localized Stern–Volmer coefficient decreased with the pressure and PC-PSP/PtTFPP had a maximum at 9 kPa. The localized Stern–Volmer coefficient of PC-PSP/PtTFPP in this pressure range was always the maximum, whereas it was the minimum for PC-PSP/Ru(dpp)${}_{3}$. Note that the preparation of PC-PSP used in this study was based on the procedure reported by Gregory et al. [41] and different from the PC-PSP used by Nagata et al. [29].

**Figure 8:**
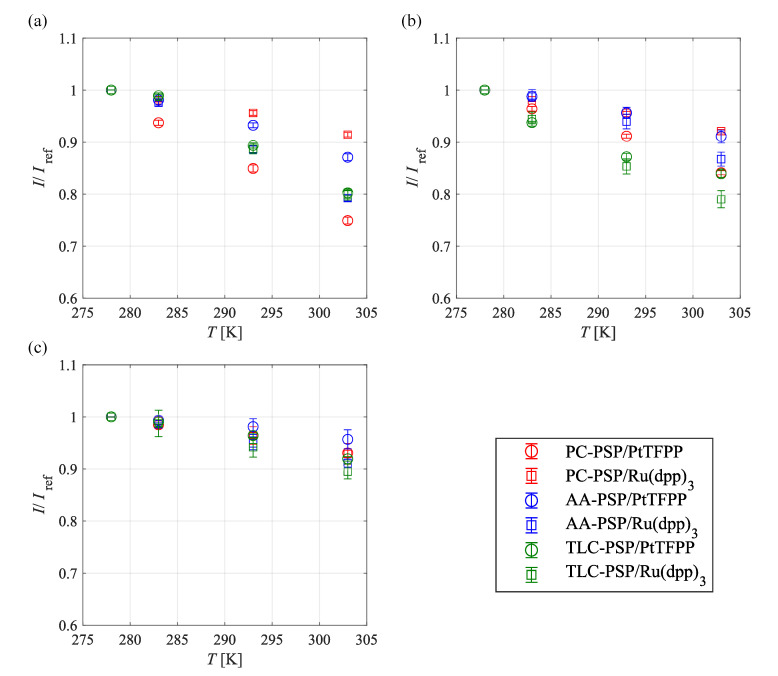
Temperature characteristics of fast-response PSPs at (**a**) 100, (**b**) 10, and (**c**) 1 kPa in the range of 278–303 K.

Temperature calibration curves for each PSP in the different pressure ranges are shown in Figure 8. Figure 8a–c shows that the PSPs with the minimum and maximum temperature sensitivity under each pressure condition were different. Figure 8a shows that the PC-PSP and AA-PSP had different temperature sensitivities that were dependent on the dye, whereas the TLC-PSP had similar temperature sensitivities at 100 kPa, regardless of the different dyes. Figure 8b shows that the influence of the different dyes on the temperature sensitivity became small under low-pressure conditions. All PSPs investigated in this study had approximately the same temperature sensitivity at 1 kPa. Lower pressure resulted in lower temperature sensitivity for all PSPs investigated because the number of collisions between dye molecules and oxygen molecules due to the increase in temperature is reduced due to the decrease in the number of oxygen molecules in the unit volume of air under low-pressure conditions.

**Figure 9:**
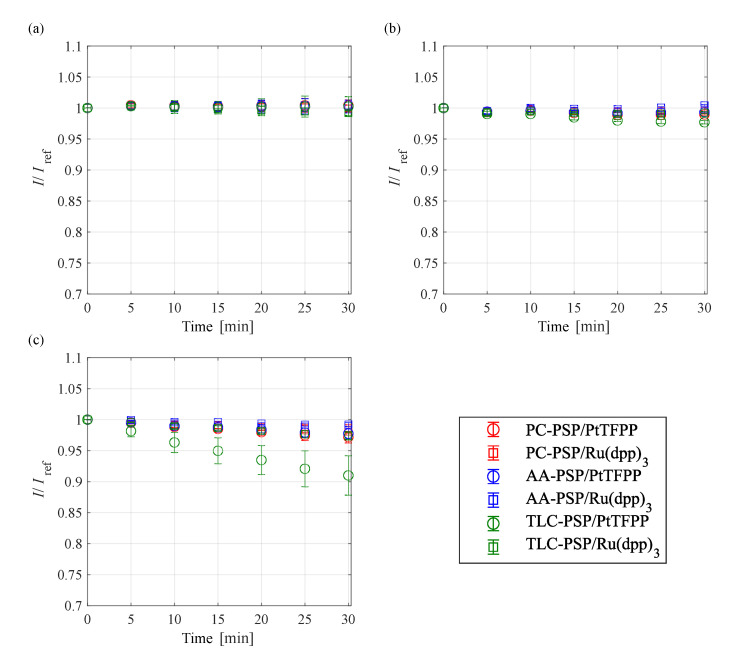
Photodegradation characteristic of fast-response PSP at (**a**) 100, (**b**) 10, and (**c**) 1 kPa at 293 K.

The photodegradation characteristic of PSPs in the different pressure ranges are shown in Figure 9. The photodegradation rate of each PSP did not change significantly at 100 kPa; however, the rate decreased as the pressure decreased. In particular, TLC-PSP/PtTFPP exhibited significant photodegradation at 1 kPa and 10 kPa. At low pressure, the number of oxygen molecules decreases, and oxygen quenching is less effective than at atmospheric pressure. The excited dye is thus likely to undergo photodecomposition, wherein the dye itself decomposes energy into radicals and atoms, rather than as luminescence. The photodegradation rate thus decreased at low pressure.

**Figure 10:**
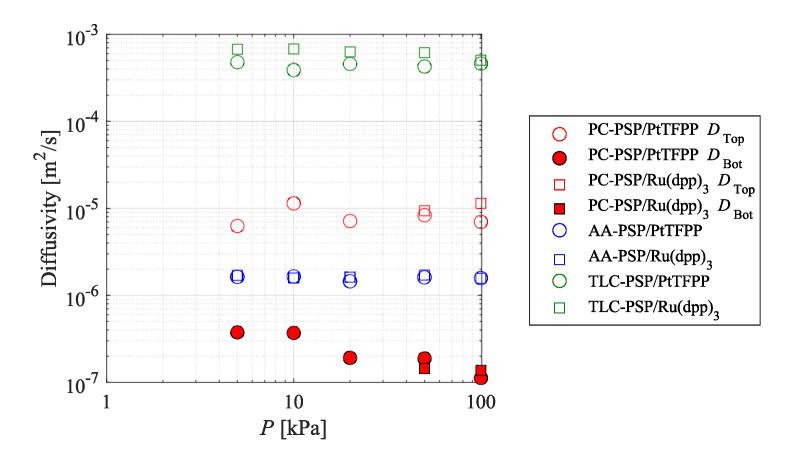
Effect of ambient pressure on the diffusivity coefficient.

Table 4 summarizes the pressure sensitivity, temperature sensitivity, and photodegradation rate of each PSP. The photodegradation rate was lower when PtTFPP was used as the dye in each binder. It is supposed that the longer lifetime of PtTFPP results in a higher probability that the excited molecules will photodecompose. Comparison of the photodegradation rates between the three binders indicated that the photodegradation rates were in the order TLC-PSP > PC-PSP > AA-PSP. The diffusivity coefficient (Figure 10), which is described later, was highest for TLC-PSP and lowest for AA-PSP. It is considered that a larger diffusivity coefficient leads to a larger number of collisions with oxygen molecules, and so photooxidation proceeds more easily.

### 3.2. Dynamic Characteristics

**Figure 11:**
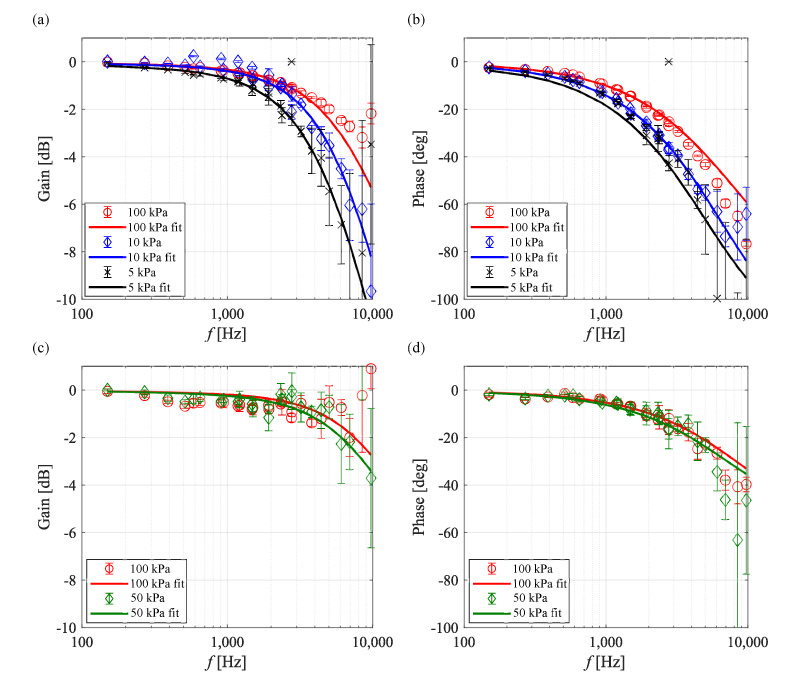
Bode plots of the PC-PSP at $T_{\mathrm{ref}}=293$ K. PC-PSP/PtTFPP: (**a**) gain and (**b**) phase. PC-PSP/Ru(dpp)${}_{3}$: (**c**) gain and (**d**) phase.

Figure 11 shows Bode plots for PC-PSP/PtTFPP and PC-PSP/Ru(dpp)${}_{3}$. The solid line is the frequency response predicted using the parameters fitted with the experimental data. As the pressure decreased, the gain and phase of PC-PSP/PtTFPP were decreased and delayed, respectively. Therefore, the response characteristics of PC-PSP/PtTFPP worsened under the low-pressure condition. The results of the frequency response model were in good agreement at all pressures.

The Bode plots of PC-PSP/Ru(dpp)${}_{3}$ fluctuated significantly, especially at 20 kPa or less, and the pressure sensitivity of PC-PSP/Ru(dpp)${}_{3}$ was quite small, 0.2%/kPa (see Figure 5 and Table 4) at 10 kPa. The frequency response of PC-PSP/Ru(dpp)${}_{3}$ could not be measured accurately because the slight pressure fluctuation at 20 kPa or less could not be captured due to the lower pressure sensitivity; therefore, the frequency response results at 20 kPa or less are not discussed. In the range of 50 kPa or more, the phase delay slightly increased as the ambient pressure decreased. However, the ambient pressure had a lesser effect on the frequency response characteristics of PC-PSP/Ru(dpp)${}_{3}$ compared with those of PC-PSP/PtTFPP.

**Figure 12:**
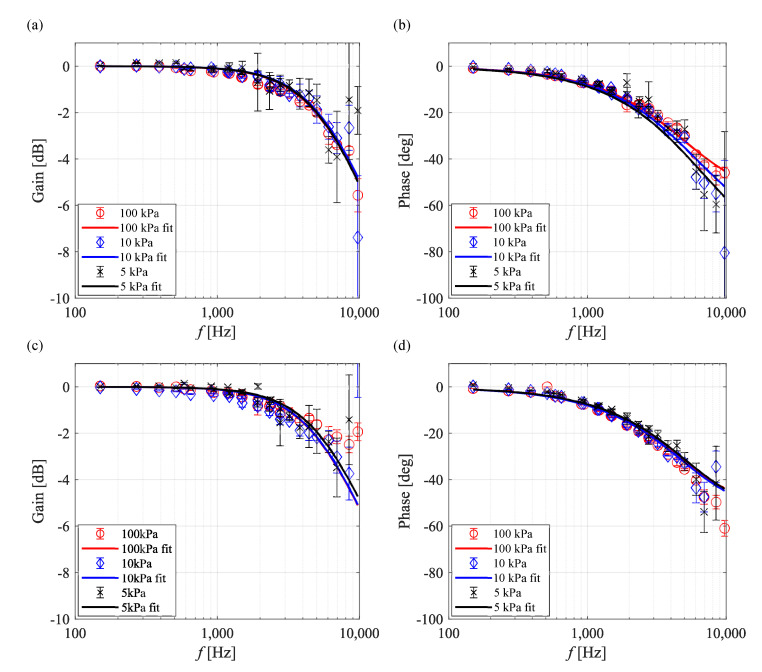
Bode plots of the AA-PSP at $T_{\mathrm{ref}}=293$ K. AA-PSP/PtTFPP: (**a**) gain and (**b**) phase. AA-PSP/Ru(dpp)${}_{3}$: (**c**) gain and (**d**) phase.

Figure 12 shows Bode plots of AA-PSP/PtTFPP and AA-PSP/Ru(dpp)${}_{3}$. The gain of AA-PSP/PtTFPP fluctuated at high frequency under low-pressure conditions, and the phase delay increased. On the other hand, the frequency response of AA-PSP/Ru(dpp)${}_{3}$ was not significantly affected by the ambient pressure. Based on the calculation formula proposed by Nonomura and Asai [35], the response of the AA-PSP could be approximated by a formula that considers oxygen diffusion in the binder and the luminescence lifetime. However, it should be noted that the calculation was performed as a model of one layer in a substance with the assumption that both the hiding factor and the thickness of the second layer were zero.

**Figure 13:**
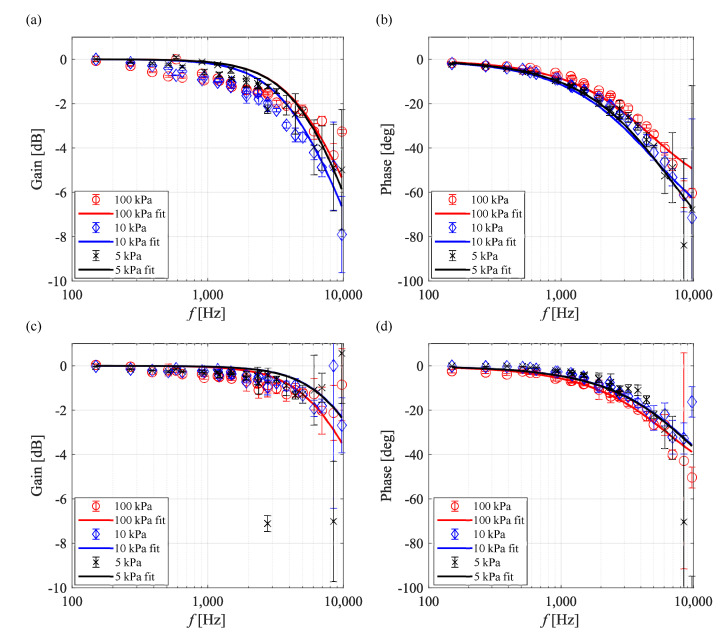
Bode plots of the TLC-PSP at $T_{\mathrm{ref}}=293$ K. TLC-PSP/PtTFPP: (**a**) gain and (**b**) phase. TLC-PSP/Ru(dpp)${}_{3}$: (**c**) gain and (**d**) phase.

Figure 13 shows Bode plots of TLC-PSP/PtTFPP and TLC-PSP/Ru(dpp)${}_{3}$. TLC-PSP/Ru(dpp)${}_{3}$ had a smaller gain and phase delay than TLC-PSP/PtTFPP at high frequency. The phase delay of TLC-PSP/PtTFPP increased slightly with a decrease in the ambient pressure, while the frequency response characteristics of TLC-PSP/Ru(dpp)${}_{3}$ were approximately independent of the ambient pressure. The decrease of the frequency response of TLC-PSP/Ru(dpp)${}_{3}$ caused by the decrease in the ambient pressure was smaller than that of PC-PSP/PtTFPP. Similar to the AA-PSP, the frequency response model with consideration of the oxygen diffusion and the lifetime at any pressure could successfully approximate the frequency response of the TLC-PSP.

The diffusivity coefficients identified for each PSP by fitting the two-layer model proposed by Nonomura and Asai [35] are shown in Figure 10. The hiding factor of the PC-PSP obtained by the gradient descent was 5.21 ×$10^{4}$ for both dyes. The closed symbols indicate the diffusivity coefficient of the bottom layer of the PC-PSP. The diffusivity coefficients of the PSPs were dependent on the binder, whereas they did not change significantly with the ambient pressure. The TLC-PSP had the highest diffusivity coefficient among all the binders, followed by the top layer of the PC-PSP, AA-PSP, and the bottom layer of the PC-PSP. The diffusivity coefficients did not change significantly due to the decrease in the pressure, except for the diffusivity coefficient of the bottom layer of the PC-PSP. On the other hand, the diffusivity coefficient for the bottom layer for the PC-PSP increased as the pressure decreased.

**Figure 14:**
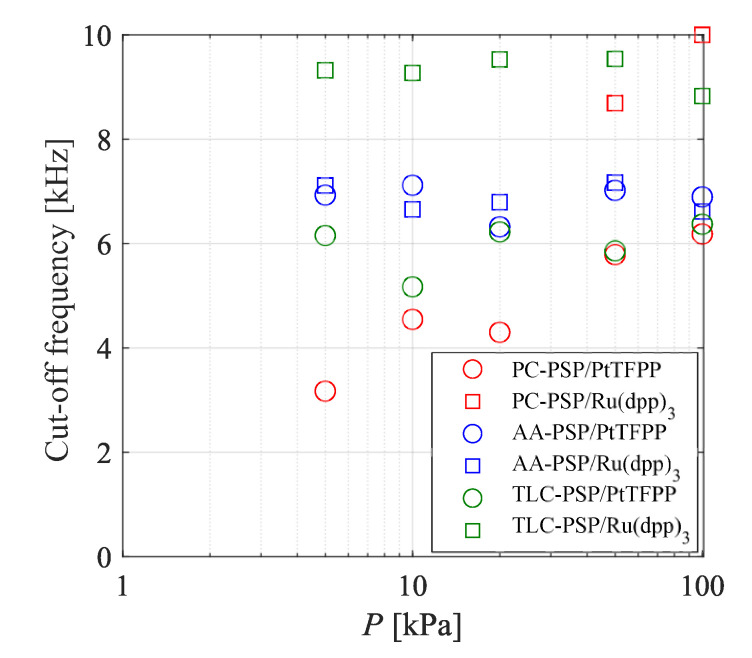
Effect of ambient pressure on the cut-off frequency.

The cut-off frequencies calculated with the frequency response model of each PSP are shown in Figure 14. The cut-off frequencies of the PC-PSP and TLC-PSP using PtTFPP were smaller than those of the PSPs with Ru(dpp)${}_{3}$, while the cut-off frequency of the AA-PSP was approximately constant, regardless of the dye. The cut-off frequencies of the TLC-PSP and AA-PSP were approximately constant, even when the pressure decreased. On the other hand, the cut-off frequency of the PC-PSP decreased with the pressure. The cut-off frequency at 5 kPa was approximately half that at 100 kPa.

**Figure 15:**
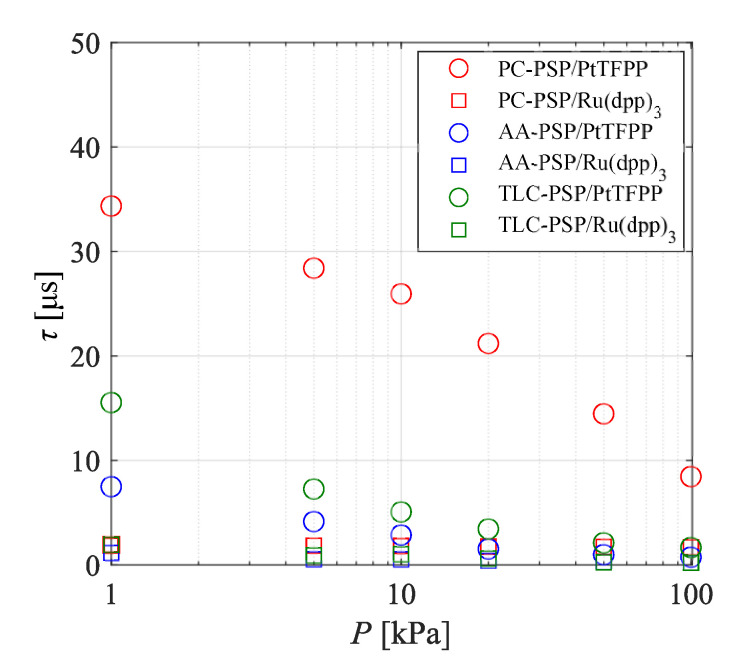
Effect of ambient pressure on the luminescence lifetime at 293 K.

Figure 15 presents the influence of the ambient pressure on the weighted mean lifetime of the PSPs. The luminescence lifetime of PC-PSP/PtTFPP was approximately 8 µs at 100 kPa, while those of the other PSPs were as short as 2 µs or less. The lower pressure led to a longer luminescence lifetime for all the PSPs investigated in the present study. In particular, the emission lifetimes of the PSPs with PtTFPP changed significantly due to changes in the ambient pressure, and the change in the amount of emission lifetime was dependent on the binder. On the other hand, there was a weak effect of the ambient pressure on the emission lifetime in the case of Ru(dpp)${}_{3}$. The emission lifetimes of PC-PSP/PtTFPP and TLC-PSP/PtTFPP, the response characteristics of which were dependent on the ambient pressure, increased by approximately 10 µs or more as the pressure decreased. The emission lifetime of AA-PSP/PtTFPP also changed with the ambient pressure, although the amount of change was as small as approximately 3 µs.

## 4. Discussion

The relationships between the structure of each binder and the diffusivity coefficient were compared first. The AA-PSP had a smaller diffusivity coefficient than the TLC-PSP and PC-PSP. The PC-PSP has particles with a diameter of 10${}^{1}$–10${}^{2}$ nm, and the TLC-PSP has particles with a diameter of 10${}^{4}$ nm and pores with a diameter of 10${}^{0}$ nm (as shown in Table 1). On the other hand, AA-PSP has a porous structure with pore sizes on the order of 10${}^{1}$–10${}^{2}$ nm. Therefore, it is considered that the diffusivity coefficient was large in the TLC-PSP and PC-PSP, which have larger-scale porous structures than AA-PSP. TLC-PSP, which is composed of silica gel with a porous structure, had a larger diffusivity coefficient because of its larger surface area.

The effect of the ambient pressure on the frequency responses of the PSPs was considered next with respect to the cut-off frequencies (Figure 14), diffusivity coefficients (Figure 10), and emission lifetimes (Figure 15). The relationship between the emission lifetime and the frequency response for each dye was investigated. The emission lifetime of AA-PSP/PtTFPP was shorter than those of PC-PSP/PtTFPP and TLC-PSP/PtTFPP. Therefore, the response characteristics of PC-PSP/PtTFPP and TLC-PSP/PtTFPP were dependent on the ambient pressure, while the influence of the ambient pressure on the response characteristics of AA-PSP/PtTFPP is considered to be small. There was less change in the luminescence lifetimes of the PSPs in response to changes in ambient pressure with Ru(dpp)${}_{3}$. Therefore, the response characteristics were not dependent on the ambient pressure in any of the samples coated with Ru(dpp)${}_{3}$.

The effect of the binder on the frequency response characteristics of the PSPs can be discussed based on the relationship between the emission lifetime and diffusivity. The diffusivity coefficient of the top layer was only considered in the case of the PC-PSP because the PSP dye is in the top layer. Although the diffusivity coefficient of the top layer did not change significantly as the pressure decreased, the cut-off frequency under low-pressure conditions decreased because the emission lifetime of the PSP with PtTFPP became longer. The emission lifetime of PC-PSP/PtTFPP was the longest of all the PSPs at any pressure, and its diffusivity coefficient was the second largest after the TLC-PSP. The frequency response of the PC-PSP can be expressed by the diffusion and the emission lifetime, even under low-pressure conditions, and the emission lifetime dominated the frequency response.

The diffusivity coefficients of the AA-PSPs were almost independent of the pressure, and the lifetimes increased as the pressure decreased, while the cut-off frequencies did not change significantly with the pressure. The lifetime of AA-PSP/PtTFPP was shorter than that of the other binders with PtTFPP under low-pressure conditions, and the difference in emission lifetime between AA-PSP/PtTFPP and AA-PSP/Ru(dpp)${}_{3}$ was the smallest under low-pressure conditions. Furthermore, the cut-off frequencies of both the AA-PSPs were almost unchanged at all pressures. The diffusivity coefficients of the AA-PSPs were the smallest of all the binders, and the cut-off frequencies did not change due to the difference in the length of the emission lifetime; therefore, the oxygen diffusion is considered to be the dominant parameter that affects the frequency response characteristics of the AA-PSPs.

The cut-off frequency of TLC-PSP/Ru(dpp)${}_{3}$ did not change significantly, while that of TLC-PSP/PtTFPP slightly decreased with the ambient pressure. The emission lifetimes of the TLC-PSPs with both dyes became longer as the ambient pressure decreased, but the diffusivity coefficients did not change. Therefore, the governing factor of the frequency response characteristics for the TLC-PSPs is the emission lifetime. This is because the diffusivity coefficient of the binder of the TLC-PSP is sufficiently large. Accordingly, the decrease in the cut-off frequency under low-pressure conditions was due to the increase in the emission lifetime.

In summary, the magnitude of the diffusivity coefficient, which is not substantially dependent on the pressure but rather on the type of binder, determines whether the cut-off frequency is dependent on the pressure. When the diffusivity coefficient is sufficiently large, the governing factor of the frequency response is the emission lifetime, and the cut-off frequency is dependent on the pressure. On the other hand, when the diffusivity is small, the governing factor of the frequency response is diffusion, and the cut-off frequency is not dependent on the pressure. The upper limit of the frequency response of each binder is determined by the lifetime of the dye in the applied state, and the diffusivity coefficient of the binder determines how slow the frequency response is. Note that the lifetime of the PSP also changes due to interference with the solvent and polymer when the dye is applied.

## 5. Conclusions

The characteristics of fast-response PSPs under low-pressure conditions were evaluated. Three representative porous binders, PC-PSP, AA-PSP, and TLC-PSP, and two types of dye, PtTFPP and Ru(dpp)${}_{3}$, were combined.

The results of static pressure calibration show that PC-PSP/PtTFPP, AA-PSP/Ru(dpp)${}_{3}$, and TLC-PSP/PtTFPP have high pressure sensitivity exceeding 4%/kPa under low-pressure conditions. PC-PSP/Ru(dpp)${}_{3}$ has low pressure sensitivity under both atmospheric and low-pressure conditions. For all PSPs, the temperature sensitivities and the photodegradation rates became lower as the ambient pressure decreased.

The results of dynamic calibration show that the frequency responses of PSPs with PtTFPP are dependent on the ambient pressure, whereas the characteristics of PSPs with Ru(dpp)${}_{3}$ are not influenced by the ambient pressure. TLC-PSP/Ru(dpp)${}_{3}$ had the highest cut-off frequency at low pressure, followed by the AA-PSPs. The cut-off frequencies of the AA-PSPs were almost constant for both the PtTFPP and Ru(dpp)${}_{3}$ dyes under both atmospheric and low-pressure conditions. Under low-pressure condition, the frequency response of the PC-PSP, AA-PSP, and TLC-PSP could be well expressed by a model that considers the luminescence lifetime and the diffusion of oxygen in the binder.

The diffusivity coefficient was almost pressure-independent and was approximately the same as the diffusivity coefficient for the same binder. The diffusivity was clarified to be larger in the order of TLC-PSP > PC-PSP > AA-PSP under low- to atmospheric pressure conditions.

Under low-pressure conditions, the emission lifetimes of all the PSPs investigated in the present study increased compared to the lifetimes under atmospheric pressure conditions. The changes in the emission lifetimes were large, in the order of PC-PSP > TLC-PSP > AA-PSP.

The governing factors for the frequency responses of the binder were clarified by comparison of the cut-off frequencies, the diffusivity coefficients, and the emission lifetimes for each dye under low-pressure to atmospheric pressure conditions. The frequency responses of all the PSPs could be expressed by the diffusion of oxygen in the binder and the emission lifetime, while pressure dependence appeared only with respect to the emission lifetime. The frequency responses were dependent on the pressure only when the diffusivity coefficient was sufficiently large, and the emission lifetime was the dominant influence in the frequency response. The results of the present study suggest that the frequency responses of PC-PSP and TLC-PSP are lifetime-dominant while the frequency responses of AA-PSP is diffusion-dominant. It is also noted that the governing factors of these PSPs may change because the state of the binder is dependent on the materials and the method of preparation.

## Figures and Tables

**Table 1:** Scale of porous binder structure.

Order of Porous Structure	PC-PSP	AA-PSP	TLC-PSP
Pore size (nm)	-	$10^{1}$–$10^{2}$	$10^{0}$
Particle size (nm)	$10^{1}$–$10^{2}$	-	$10^{4}$

**Table 2:** Sample preparation conditions.

Binder	Dye	Solvent	Dye Coating Type	Thickness (µm)
PC-PSP	PtTFPP	Toluene	Spray	57.5
	Ru(dpp)${}_{3}$	Hexane		61.5
AA-PSP	PtTFPP	Toluene	Dipping	10.0
	Ru(dpp)${}_{3}$	Dichloromethane		10.2
TLC-PSP	PtTFPP	Toluene	Spray	177.6
	Ru(dpp)${}_{3}$	Dichloromethane		154.8

**Table 3:** Initial values of the frequency response model.

Binder	Dye	Thickness (µm)		Diffusivity (m${}^{2}$/s)		Hiding Factor *c* (1/m)
		Top	Bottom	Top, $D_{\mathrm{Top}}$	Bottom, $D_{\mathrm{Bot}}$	
PC-PSP	PtTFPP	20.4	37.1	3.29 ×$10^{-5}$	5.15 ×$10^{-9}$	5.21 ×$10^{4}$
	Ru(dpp)${}_{3}$	21.0	41.5	6.98 ×$10^{-6}$	1.12 ×$10^{-8}$	
AA-PSP	PtTFPP	10.0	-	1.54 ×$10^{-6}$	-	0
	Ru(dpp)${}_{3}$	10.2				
TLC-PSP	PtTFPP	177.6		4.54 ×$10^{-4}$		
	Ru(dpp)${}_{3}$	154.8				

**Table 4:** Summary of static characteristics for PC-PSP, AA-PSP, and TLC-PSP.

Binder		PC-PSP		AA-PSP		TLC-PSP	
Dye		PtTFPP	Ru(dpp)${}_{3}$	PtTFPP	Ru(dpp)${}_{3}$	PtTFPP	Ru(dpp)${}_{3}$
$S_{P}$ (%/kPa)	1–10 kPa	6.1	0.20	2.9	4.9	4.5	2.2
	10–100 kPa	0.55	0.085	0.41	0.59	0.54	0.50
$S_{T}$ (%/K)	1 kPa	0.28	0.30	0.18	0.37	0.33	0.44
	10 kPa	0.63	0.31	0.39	0.60	0.51	0.77
	100 kPa	0.97	0.35	0.55	0.89	0.89	0.89
$I_{d}$ ($10^{-2}$%/min)	1 kPa	−9.6	−5.5	−7.6	−2.8	−30	−8.9
	10 kPa	−3.2	−0.71	−2.0	1.6	−7.4	−2.4
	100 kPa	0.87	−0.71	0.44	1.1	0.41	−3.4

## Data Availability

Data sharing not applicable.
